# [Retracted] Puerarin attenuates glucocorticoid-induced apoptosis of hFOB1.19 cells through the JNK- and Akt-mediated mitochondrial apoptotic pathways

**DOI:** 10.3892/ijmm.2026.5836

**Published:** 2026-04-20

**Authors:** Dongdong Yu, Shuai Mu, Danyang Zhao, Guangbin Wang, Zhiguang Chen, Hongfei Ren, Qin Fu

Int J Mol Med 36: 345-354, 2015; DOI: 10.3892/ijmm.2015.2258

Following the publication of the above paper, a concerned reader has drawn to the Editor's attention that, regarding the TUNEL assay data shown in Fig. 4A on p. 348, the images shown for the 'control' and 'puerarin 0M' experiments contained an overlapping section, such that data which were intended to show the results of differently performed experiments were apparently derived from the same original source. In addition, regarding the flow cytometric plots shown in Fig. 10A on p. 351, the 'SP600125+DEX' and 'LY294002+DEX' data panels were also strikingly similar. Upon investigating the data in this paper independently in the Editorial Office, it also came to light that certain of the western blot data in Fig. 8A and C were overlapping with data included in Fig. 9A and C, and moreover, western blot data featured in Fig. 5 subsequently appeared in an article featuring a pair of the same authors in the journal *Neural Regeneration Research*.

In view of the large number of apparent errors that were made in assembling various of the data in the figures reported above, and given the re-use of some of the data in a subsequently published paper that featured a pair of the authors on the above paper, the Editor of *International Journal of Molecular Medicine* has decided that this paper should be retracted from the Journal on account of a lack of confidence in the presented data. The authors were asked for an explanation to account for these concerns, but the Editorial Office did not receive a reply. The Editor apologizes for any inconvenience caused.

